# Video-Supported Remote Cognitive Assessment in General Practice—A Pilot Mixed-Method Study on Usability, Acceptability and Feasibility

**DOI:** 10.3390/healthcare14111452

**Published:** 2026-05-25

**Authors:** Alexa Holfelder, Esther Brill, Rachid Guerchouche, Minh Tran-Duc, Jacob Lahr, Stefan Klöppel

**Affiliations:** 1 University Hospital of Old Age Psychiatry and Psychotherapy, University of Bern, 3012 Bern, Switzerland; esther.brill@unibe.ch (E.B.); jacob.lahr@upd.ch (J.L.); stefan.kloeppel@unibe.ch (S.K.); 2 Graduate School for Health Sciences, University of Bern, 3012 Bern, Switzerland; 3 Cognition Behavior Technology (CoBTeK) Lab, Université Côte d’Azur, 06100 Nice, France; rachid.guerchouche@inria.fr (R.G.); tdminh2110@gmail.com (M.T.-D.); 4 Institut National de Recherche en Informatique et en Automatique (Inria), 06902 Valbonne, France

**Keywords:** digital health, telemedicine, elderly, remote cognitive assessment, dementia screening, general practice, primary care integration

## Abstract

**Highlights:**

**What are the main findings?**

- Remote Cognitive Assessment (RCA) is technically feasible in primary care and shows acceptance among older patients and general practitioners.
- Neuropsychologists identified major limitations in usability and diagnostic depth, indicating important challenges for routine clinical implementation.

**What are the implications of the main findings?**

- RCA may offer a complementary approach in underserved and rural areas, but high patient satisfaction alone is insufficient for routine clinical adoption.
- Future development must prioritize diagnostic confidence and clinician workflow usability before integration into routine primary care can be established.

**Abstract:**

Background/Objectives: Access to specialists and diagnostic resources continues to limit differential diagnosis of cognitive impairment in primary care. This pilot study examined the feasibility, usability, and clinical integration of a digitally supported Remote Cognitive Assessment (RCA) model embedded in general practice settings. Methods: A mixed-method design was used, combining structured quantitative surveys from patients (*n* = 10; mean age = 77.03; SD = 14.1) and neuropsychologists (10 RCAs completed by three neuropsychologists) with qualitative interviews from general practitioners (GP; *n* = 4). Patients were assessed remotely via a secure videoconference system operated by trained neuropsychologists. Assessments were conducted in the GP’s office, supported by local staff, to facilitate the process. Results: Patients reported high satisfaction with audio (M = 8; SD = 2.28) and video quality (M = 9.17; SD = 1.17) and expressed a strong willingness to recommend RCA (M = 8.83; SD = 1.17) on a 10-point Likert scale. Despite moderate scores for perceived simplicity (M = 5; SD = 3.41) and effectiveness (M = 5.83; SD = 2.14), overall acceptance (M = 8.33; SD = 0.82) was favorable, especially given the older age of participants. Neuropsychologists rated technical functionality positively (audio quality M = 8.17; SD = 1.18; video quality M = 8; SD = 1.67) but raised concerns about clinical utility and diagnostic depth (effectiveness M = 2.83; SD = 2.71). GPs highlighted the benefits of local facilitation, early screening, and improved access to specialist input while also noting space limitations, communication gaps, and the need for sustainable infrastructure. Conclusions: The RCA model was well accepted by patients and GPs, and technically feasible for neuropsychologists. However, neuropsychologists reported important reservations regarding usability and effectiveness. The results suggest an important mismatch between patient satisfaction and clinical confidence and RCA cannot yet be recommended for routine clinical implementations based on patient acceptability alone. This model holds promises for hybrid cognitive care, particularly in underserved or rural areas, but future development must prioritize diagnostic confidence and clinician workflow usability before scalable integration into rural cognitive care pathways can be established.

## 1. Introduction

Healthcare systems worldwide face the dual pressures of population aging and an increasing burden of patients with complex needs, compounded by persistent regional disparities in care provision [1,2]. These challenges are particularly acute in rural and remote areas, where access to affordable, high quality, and specialized healthcare services remains limited [3,4]. In Switzerland, nearly half of all municipalities are classified as rural “hard-to-serve” areas, highlighting regional disparities in care provision [5]. One critical area affected by these disparities is the detection and management of cognitive decline [6,7]. However, low diagnostic rates cannot be attributed to access barriers alone. Delayed diagnosis is a multifactorial problem, influenced not only by limited specialist availability and travel burden, but also low symptom awareness, stigma, reduced insight, reluctance to seek assessments, and therapeutic nihilism among patients, families, and healthcare professionals [8,9]. Specialized memory clinics, which are essential for diagnosing and treating cognitive-decline-related disorders like Alzheimer’s disease (AD) and other forms of dementia, are scarce outside urban centers [6]. Currently, over 150,000 Swiss residents live with dementia, yet only half receive an early diagnosis, reflecting a combination of structural, individual, and clinical barriers, including difficulties accessing specialist care [10,11,12]. Owing to demographic aging and improved case detection, this number is projected to double within 30 years, imposing a significant social and economic burden [10,13]. These access barriers are even more pronounced in larger OECD (Organization for Economic Co-operation and Development) countries [14]. A recent scoping review found that residents of rural and remote areas face significant distance decay effects, with healthcare access, including specialized memory clinics, decreasing sharply even at relatively short travel times of 30 to 60 min [15].

Differential detection of cognitive impairment is essential for effective AD treatment. In-person assessment at specialized memory clinics is the clinical gold standard and offers important advantages, particularly for older adults with complex needs [16]. Face-to-face encounters allow clinicians to build a rapport, observe subtle behavioral and neurological signs, involve caregivers directly, and provide a personal and reassuring clinical environment [17]. The experience from the COVID-19 pandemic also highlighted that, despite the usefulness of remote modalities, many patients, caregivers, and clinicians value a return to in-person consultations and the interpersonal aspects of face-to-face care [18]. Nevertheless, traditional memory clinic protocols rely on lengthy, face-to-face and paper-and-pencil cognitive assessments administered by trained clinicians [16]. Those procedures can be resource-intensive for the memory clinics and for patients living far from specialist centers; this may create additional barriers, including travel burden and travel-related fatigue and stress. Remote Cognitive Assessment (RCA) offers a promising complementary approach by enabling selected components of cognitive evaluations to be delivered closer to patients’ home while maintaining specialist involvement. For some patients, the main potential benefit of this model is reduced travel burden and improved access to specialist input [19]. Despite the promise, many existing RCA solutions assume patient proficiency with computers and videoconferencing tools, an unrealistic expectation for some older adults, especially those with cognitive impairments. While internet usage among Swiss adults aged 65+ has reached 89%, only 10% demonstrate advanced digital skills, with capabilities declining sharply in those over 85 [20]. This digital divide, particularly pronounced in rural areas and among individuals with lower income or educational levels, underscores the need for user-friendly, supportive technologies that do not rely on high digital literacy.

Recent advances in computerized cognitive testing, automatic speech and image analysis, and remote sensing have enabled more objective and scalable evaluation of patients’ cognitive, behavioral, and emotional status [21]. Furthermore, studies demonstrate high concordance between virtual and in-person dementia assessments [22,23,24]. However, these investigations have typically focused on only a few cognitive tasks rather than comprehensive battery or used generic videoconferencing platforms (e.g., Microsoft Teams). Thus, although prior studies have supported the technical feasibility and psychometric reliability of RCA, less is known about its implementation in routine primary care settings, particularly when assessments are delivered as part of a regional memory clinic referral pathway.

To address this gap, our collaborators in France at the Institut National de Recherche en Informatique et en Automatique (Inria), Valbonne, France, developed a dedicated videoconference (VC) system capable of delivering a full neuropsychological assessment, encompassing multiple cognitive tests, psychiatric evaluation, and medical history interviews, via two tailored interfaces [24]. The clinician interface provides test menus, timing controls, scoring algorithms, and real-time speech/video recording. The patient interface presents only relevant test content in a highly simplified format. The platform has been evaluated by Zeghari et al. [24] in a mobile-clinic model, in which the system was installed in a van parked near the participants’ home and provided important evidence for the reliability of the system. However, it did not examine how the VC system could be embedded into routine primary care, where implementation depends on local infrastructure, staff roles, scheduling, communication with specialists, and compatibility with general practice workflows. The present pilot mixed-method study therefore addresses the implementation gap of whether the RCA model using the VC system can be feasibly and acceptably delivered in GP practices in rural areas of the greater Bern region, Switzerland. Accordingly, this study provides preliminary feasibility evidence, with a particular focus on comparing stakeholder perspectives. The objectives are to (1) assess older patients’ and neuropsychologists’ experiences of RCA, (2) explore the perspectives of general practitioners regarding clinical workflow, usability, and perceived benefits and limitations, and (3) identify practical, technical, and implementation-related factors relevant to the future use of RCA in rural health care settings.

## 2. Materials and Methods

### 2.1. Equipment

#### The Videoconference (VC) System

**Figure 1:**
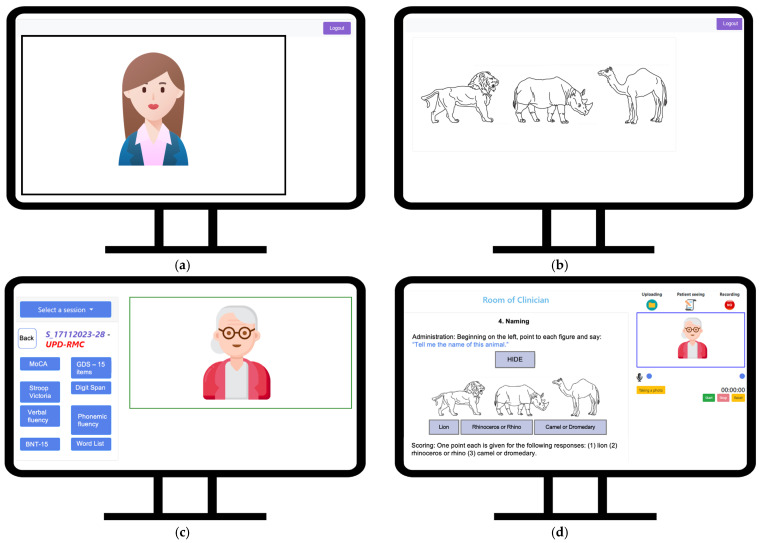
Schematic representation of the VC system. The patient screen either shows (**a**) the neuropsychologist or (**b**) test materials (e.g., objects of the MoCA [25]). The neuropsychologist screen either shows (**c**) the patient and a selection of several neuropsychological tests or (**d**) test instructions, scoring options and the patient.

The developed VC system, as shown in Figure 1, is a fully web-based platform specifically designed for the diagnosis, screening, and monitoring of cognitive disorders.

Built on the latest advancements in information and communication technologies, the platform facilitates remote care with any internet browser, eliminating the need for specialized software installation. It enables a secure and direct connection between clinicians and patients, each accessing the system with personal login credentials. The platform incorporates two distinct user interfaces tailored to the differing needs of clinicians and patients. The patient interface is intentionally simplified to allow for use by individuals with limited or no experience using computers or internet browsing. During the assessment, patients can remain entirely passive, interacting only through verbal communication. However, if comfortable, they may also engage with the platform via simple actions such as clicking buttons, checking boxes, or selecting images. In contrast, the clinician interface is more advanced and supports the full functionality required for administering and managing assessments.

The system allows clinicians to conduct clinical interviews via real-time video and/or audio communication, with the option to record the sessions. It includes over 20 validated and widely used neuropsychological tests and clinical rating scales, available in German and French. Throughout the assessment process, clinicians can share visual, auditory, or textual content with the patient as needed. Test results are automatically scored, securely stored, and can be reviewed within the platform. Furthermore, all test sessions, including speech and video from the patient side, can be recorded and subsequently accessed by the clinician for detailed review or further diagnostic evaluation. Originally developed in France, for the current study, we adapted the VC system for use in Switzerland by translating the full test battery and interface into German and deploying a dedicated version of the platform hosted on Swiss servers. This ensured compliance with national data protection regulations. Unlike general-purpose VC tools such as Microsoft Teams, this platform is specifically developed to support comprehensive RCA. It ensures standardized testing conditions, accommodates a wide range of patient abilities, and provides a secure infrastructure for data handling. The dedicated design enhances both clinical reliability and user accessibility, positioning it as a robust solution for delivering specialist cognitive care remotely.

### 2.2. Participants

For this pilot study, we included 10 patients (mean age = 77.03, SD = 14.1; Female = 4, Male = 6) that were referred by one of our cooperating general practices in the greater Bern region, Switzerland. The sample size was determined pragmatically according to the pilot nature of this study, the number of participating GP practices, and the feasibility of conducting RCA within the study period. Patients were not recruited consecutively from all GP attendees. Instead, recruitment followed a pragmatic, practice-based approach during routine consultations. GPs identified patients with suspected age-or disease-related cognitive decline or responded to cognitive concerns raised by patients. If the GP considered further clarification appropriate and the patient agreed, the option of RCA was introduced and referral to the IDMC Bern was initiated. This approach reflects routine clinical decision making. Participating GPs were required to be located in the greater Bern area and to provide an appropriate room for conducting the RCA. In total, four GPs participated in this pilot study. Participant recruitment took place between July 2024 and June 2025. The inclusion criteria were: living in the greater Bern region, age- and/or disease-related cognitive decline, ability to understand and speak German, above 55 years. Exclusion criteria: significant vision and auditory problems which would impact ability to perceive and understand the clinician. Informed consent was obtained from all participants.

### 2.3. Procedure and Protocol

**Figure 2:**
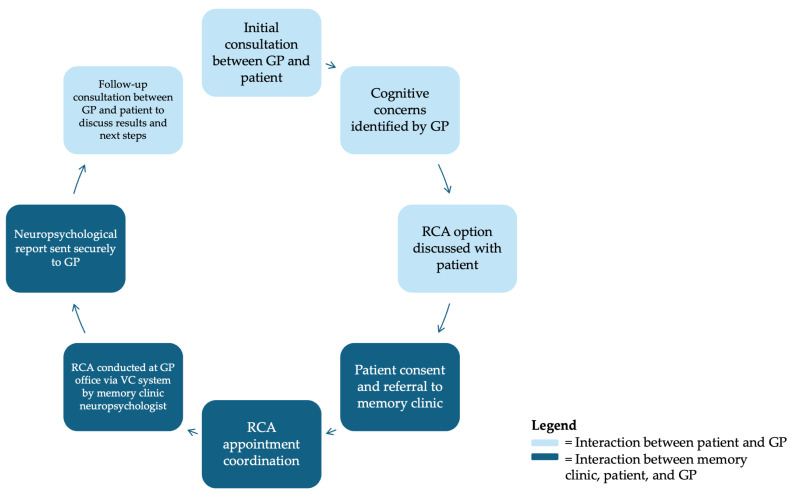
Workflow of the RCA process. This schematic diagram illustrates the stepwise protocol for implementing RCA in GP. Box color indicates the interaction type: light blue boxes represent interactions between patient and GP, while dark blue boxes represent interaction between memory clinic, patient, and GP. The process begins with a standard GP consultation during which cognitive concerns are identified. Upon agreement, the patient is referred to the IDMC and registration for RCA is initiated. Administrative coordination and scheduling are managed by the research team. RCA is conducted at the GP’s office connected with an NP from the IDMC using the VC system. Following the session, structured feedback is collected. NP compiles a report, which is securely transmitted back to the GP. A follow-up GP consultation is then held to communicate results and determine appropriate next steps. GP, general practitioner; RCA, remote cognitive assessment; VC system, videoconference system.

Prior to the start of participant recruitment, preparatory meetings were held with participating GPs and their practice teams to introduce the project, align on procedural steps, and set up the necessary technical infrastructure. This pilot study was conducted in community-based GP practices located in the greater Bern area, Switzerland, thereby reflecting routine clinical pathways and reducing potential referral and ecological bias. These meetings included detailed discussions on the study objectives, patient inclusion criteria, data protection procedures, and the use of the VC system. Technical compatibility was verified at each study site, and all necessary equipment was tested to ensure smooth operation during assessments. Following the initial setup, a comprehensive training session was conducted with the GP office staff. This training covered the full workflow of the RCA including patient referral, platform access, scheduling, and support during the testing. Emphasis was placed on ensuring that the staff felt confident in guiding patients and assisting with technical aspects, where needed. In addition, each participating practice received an instruction manual outlining the assessment workflow and step-by-step procedures for managing technical problems during testing. If technical difficulties occurred, the study team was available by telephone to provide real-time support. Once the preparatory phase was completed, the patient assessment process followed a standardized sequence (Figure 2).

Recruitment occurred during routine GP consultations in which signs of cognitive decline were observed, supporting a naturalistic inclusion process and reducing selection bias. In cases where further clarification was deemed necessary, the GP introduced the option of an RCA using the VC system. If the patient agreed, the GP referred them to the Interdisciplinary Memory Clinic (IDMC) Bern, Switzerland, and initiated the registration process.

The research staff then coordinated the administrative steps and scheduled the RCA appointment. Communication with patients regarding the appointment details and questions was managed primarily via telephone, thereby avoiding reliance on digital communication tools and reducing access bias related to digital literacy. Importantly, the assessment was not conducted at the patient’s home but took place in the referring GP’s office. This approach allows the patient to remain closer to home in a locally accessible healthcare setting and reduces travel time but does not require any prior knowledge of technology from the patient side nor home-internet access while still gaining access to specialist care. The GP practice setting was chosen to ensure a standardized technical setup, stable internet connection, and the availability of on-site procedural support. Patients were not required to have a study or trial partner. Instead, trained GP practice staff introduced the platform, explained the procedure, and remained available on site to assist patients if they experienced difficulties operating the system. This step helps reduce selection bias by not limiting participation to individuals with reliable internet connection at home or higher levels of digital literacy.

The RCA itself was administered by a trained neuropsychologist from the IDMC Bern using the secure, web-based VC platform. The neuropsychologists were also trained in the workflow and how to use the platform, conduct the test and report the results back to the GP. The assessment session included a brief clinical interview followed by a series of standardized cognitive screening tests included in the Consortium to Establish a Register for Alzheimer’s Disease (CERAD) neuropsychological test battery [26]. The following tests were conducted: Montreal Cognitive Assessment (MoCA) [25], Victoria Stroop Test [27], Verbal Fluency [27], Boston Naming Task (BNT-15) [28], Geriatric Depression Scale (GDS-15) [29], Digit Span Forward and Backward [30], Phonematic Fluency [30], CERAD Wordlist Memory Test [26], and Trail Making Test A and B [31]. The online platform allowed for real-time video and/or audio communication and offered the option to record the session and take screenshots for quality assurance and diagnostic verification.

After the assessment, both the neuropsychologist and the patient completed structured online questionnaires to evaluate the experience, usability, and acceptability of the remote assessment process. A comprehensive report, including test results and clinical impressions, was prepared by the neuropsychologist and securely transmitted back to the referring GP. Finally, the GP reviewed the results and scheduled a follow-up consultation with the patient to discuss the findings. The GP explained the outcomes of the assessment and determined the appropriate next steps, which could include additional diagnostics, initiation of treatment, or ongoing monitoring depending on the patient’s needs.

To further evaluate the feasibility, integration, and acceptability of the VC-based assessment model, structured qualitative interviews were conducted at the conclusion of the pilot phase with participating GPs. These interviews explored their experiences, perceived benefits and challenges, and recommendations for future implementation and scale-up.

### 2.4. Data Collection and Availability

This study was reviewed by the local Ethics Committee in Bern (identifier Req. 2022-00738) and categorized as not falling under the Human Research Act [32]. The assessments were performed remotely by neuropsychologists from the IDMC Bern; the VC system recorded scores, videos, and screenshots stored on a dedicated secured server, complying with healthcare data hosting regulations. Highly sensitive data were collected and due to privacy reasons, the data and analytical code supporting the findings of this study are available from the corresponding author on reasonable request. All data collected in this study was anonymized and no identification of individual participants in the data or manuscript is possible. Collection, processing, and storage procedures for this study strictly adhered to Swiss legal and ethical frameworks, including the Swiss Federal Act on Research involving Human Beings (Human Research Act, HRA) and the new Federal Act on Data Protection (nFADP). Participants were not compensated for participating.

### 2.5. Acceptability Evaluation

All participants were asked to answer a questionnaire (Supplementary Material File S1) on the acceptance of the VC system, including questions with a response ranging from 1 to 10, where 1 = I strongly disagree and 10 = I strongly agree. This questionnaire was adapted from the User Satisfaction and Ease of use (USE) questionnaire [33] by retaining and rewording selected items from the original usefulness, ease of use, and satisfaction domains for the RCA context, including simplicity of use, perceived usefulness and effectiveness, overall satisfaction, and willingness to recommend the system. Additional study-specific items were added to assess audio and video quality, technical problems, and communication and interaction quality. Additionally, the questionnaire also included open-ended questions regarding positive and negative features and open feedback. Each patient completed an acceptability scale immediately after the RCA. Also, the neuropsychologist completed a questionnaire (Supplementary Material File S2) on the acceptance of the VC system following the same structure. Additionally, neuropsychologists and patients completed the German version of the System Usability Scale (SUS; Supplementary Material File S3) [34], which is a 10-item questionnaire scored on a 5-point Likert scale (1 = Strongly disagree and 5 = Strongly agree) and provides a measure of a user’s perception of the usability of a system. The SUS is a single score, ranging from 0 to 100, representing a composite measure of the overall usability of the system. The average score (at the 50th percentile) is 68, meaning a score above 68 is above average and one below 68 is below average. All questionnaires were administered online immediately after the RCA.

To complete the quantitative data and gain deeper insight into user experiences, semi-structured qualitative interviews were conducted with the participating GPs following the completion of the testing phase. The interviews were conducted by the first author A.H. These interviews explored several domains, including (1) overall impression of the RCA, (2) technical usability, and (3) practical challenges during the assessments. Further topics included (4) perceived differences compared to in-person assessments, (5) communication and collaboration between primary care and specialist teams, (6) patient reactions to the remote format, (7) potential for improvement and prospects, and (8) open remarks. Particular attention was paid to identifying potential barriers to implementation, evaluating the feasibility of conducting specific cognitive tests remotely and understanding which patient groups may be suitable for this approach. Interviewees were also asked to provide feedback on how the procedure and technical infrastructure could be improved and whether they would recommend continued use of the system in routine practice. The qualitative findings served to contextualize and enrich the quantitative acceptability data and informed potential refinements of the RCA for future use in primary care settings.

Because stakeholder groups interacted with the RCA model in different ways, different evaluation methods were used. Patients and neuropsychologists completed quantitative questionnaires because they directly participated in or administered each RCA session and could therefore evaluate the VC system immediately after use. In contrast, GPs were primarily involved in identifying eligible patients, initiating referrals, providing the local assessment setting, supporting implementation, and discussing results with patients after the assessment. Their role concerned clinical workflow and integration into primary care rather than direct test administration. Therefore, GP perspectives were collected through semi-structured qualitative interviews to capture implementation experiences, perceived barriers, and recommendations for future use.

### 2.6. Analysis

Statistical analyses were performed using R Studio, version 4.5.1 2025-06-13 [35]. A *p*-value of 0.05 was considered statistically significant. Descriptive statistics (mean, standard deviation) were used to describe demographics. Mean, median and standard deviations were computed for the user experience questionnaires. SUS scores were calculated according to standard procedures [34]. Each item’s score contribution will range from 0 to 4. For odd-numbered items, 1 was subtracted from the responses, while for even-numbered items, responses were subtracted from 5. The adjusted scores were summed and multiplied by 2.5 to obtain a total score ranging from 0 to 100. Due to the pilot nature of this study and small sample size, quantitative comparison findings were interpreted primarily descriptive. A series of non-parametric tests (Mann–Whitney U) were performed only as supplementary indicators and were not interpreted as confirmatory evidence of group differences. *p*-values should be interpreted with caution and as indicators of trends rather than definitive population effects.

Inductive thematic analysis was conducted on the interview transcripts using MAXQDA version 24.10 (VERBI Software). Interviews were audio-recorded and transcribed verbatim prior to analysis. Codes were generated directly from the data in an iterative process and subsequently grouped into overarching themes based on patterns and conceptual similarities. The analysis was conducted in two stages: an initial round of open coding, followed by a second round in which codes were reviewed, refined, and organized into higher-level themes. Coding was performed by two researchers and coding disagreements were discussed until a consensus was reached. A coding framework was developed and is included as Supplementary Material File S4. Data saturation was considered during the analysis; after the final interview, no substantially new themes were identified, suggesting that thematic saturation had been reached. Participant validation was conducted by sharing a summary of emerging themes and interpretations with the interviewees and by asking them if the themes accurately reflect their perspectives and whether perspectives were missing or misinterpreted. A reflexive approach was adopted throughout this study. The researchers recognized that their neuropsychological and psychological background could support interpretation of the data while also introducing a potential bias and shaping how participants’ responses were understood. To reduce the risk, the interview guide was structured to encourage balanced responses, including both positive experiences and perceived limitations or concerns, and an ongoing discussion among researchers regarding coding decisions, interpretation of themes, and potential influence of researchers’ assumption on the analysis was carried out. GenAI (GPT 5.3) has been used to improve sentence structure, refine wording, and to improve code for analysis. It was not used to generate codes, conduct analysis, or interpret results. All GenAI-assisted edits were reviewed by the authors to ensure that the meaning was preserved.

## 3. Results

### 3.1. Quantitative Results

#### 3.1.1. User Experience Patients

In total, 15 potential patients were identified during routine consultations. Two did not meet the inclusion criteria (*n* = 2 too young), three declined participation, and ten (mean age = 77.03, SD = 14.1) were ultimately included. For all patients, all data are available for analysis (see Table 1 for demographics and Supplementary Material File S5 for patient and data flow diagram).

The quantitative results suggest acceptability with the system. Patients rate the video (M = 9.17, SD = 1.17) audio quality (M = 8.00, SD = 2.28) very highly. The user interface was also rated positively (M = 7.67, SD = 1.51), suggesting that most patients found the platform visually and functionally accessible. In contrast, ratings for simplicity and effectiveness compared to an in-person assessment were more moderate, with mean scores of 5.00 (SD = 4.31) and 5.83 (SD = 2.14), respectively, indicating variability in user experiences regarding ease of use and perceived utility of the system. The large standard deviations indicate variability in patient experience, suggesting that some participants found the system easier to use and more effective than others. Notably, technical problems were reported infrequently (M = 1.67, SD = 0.82), which suggested only a few disruptions during the sessions. Although individual technical support needs were not systematically documented, patients did not report major difficulties regarding navigating the system to the study team. Importantly, overall satisfaction with the RCA was high (M = 8.33, SD = 0.82), and participants expressed a strong willingness to recommend the procedure to others (M = 8.83, SD = 1.17). Furthermore, the SUS yielded a mean score of 70.4 (SD = 19.7), indicating above-average perceived usability from the patient’s perspective. These results suggest that RCA was acceptable among older adults with cognitive difficulties, while variability in simplicity and perceived effectiveness indicates that patient experience was not uniformly positive.

#### 3.1.2. User Experience Neuropsychologists

A total of 10 assessments were conducted by three neuropsychologists. Following each assessment, the corresponding neuropsychologist completed both the SUS and the user experiences questionnaire. In total, 10 datasets were included in the analysis. The results from the neuropsychologists suggest a more cautious appraisal of the VC system’s performance compared to the patient ratings, with moderate scores across most evaluated domains. Audio and video quality were rated relatively high, with mean scores of 8.17 (SD = 1.17) and 8.00 (SD = 1.67), respectively, indicating that the technical infrastructure for communication was generally stable and functional. However, the simplicity of the system (M = 3.33, SD = 1.86) and the user interface (M = 5.67, SD = 2.16) were rated lower, suggesting room for improvement in terms of usability from the clinician’s perspective. Technical problems received a moderate rating (M = 5.67, SD = 2.56), indicating that occasional disruptions or usability issues were encountered. Ratings for communication (M = 3.83, SD = 3.06) and interaction with patients (M = 6.67, SD = 1.97) varied, with the latter suggesting that direct engagement with patients was feasible but not fully comparable to in-person settings. Importantly, effectiveness compared to an in-person assessment and reflecting the perceived clinical value and diagnostic utility of the RCA was rated low (M = 2.83, SD = 2.71), highlighting substantial concerns regarding the ability to administer a comprehensive neuropsychological assessment remotely. This was also reflected in the overall satisfaction score (M = 4.33, SD = 2.50) and the willingness to recommend the system to others (M = 4.67, SD = 1.63), both of which were moderate to low. Additionally, the SUS score averaged 51.7 (SD = 20.9), indicating below-average usability perceptions and reinforcing the need for improved clinician-centered design. These findings suggest that while the technical delivery of the VC system is reliable, improvements are needed in the interface design, test administration procedures, and clinical workflow integration to enhance clinician satisfaction and ensure diagnostic validity in remote settings.

#### 3.1.3. Comparison

**Figure 3:**
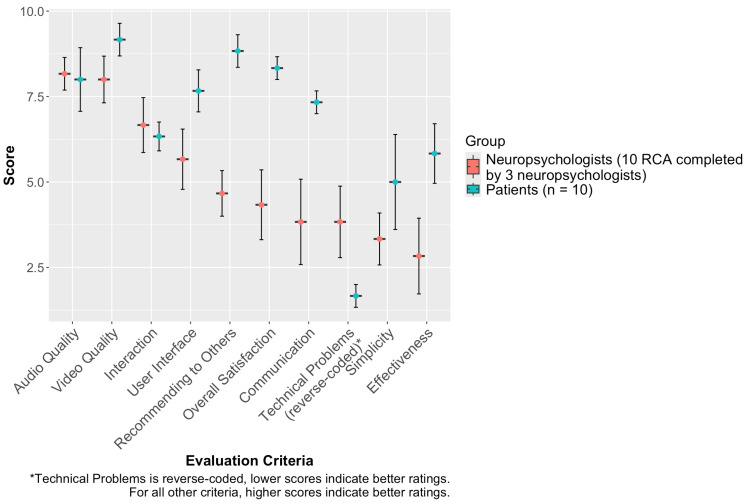
Side-by-side comparison of user experience ratings from patients and neuropsychologists during remote cognitive assessments. The figure displays mean and standard deviation of ratings for each evaluation criterion, separated by rater group. Discrepancies highlight differing perspectives on usability and effectiveness of neuropsychologists and patients. Higher values indicate more favorable ratings, except for technical problems, where higher scores reflect more frequent issues.

Given the pilot nature of this study, group comparisons are interpreted descriptively. Neuropsychologists (M = 3.44; SD = 2.5) rated overall satisfaction lower than patients (M = 8.33; SD = 0.82), and their willingness to recommend the RCA to others was also reduced (neuropsychologists: M = 4.67, SD = 1.63; patients: M = 8.83, SD = 1.17). These results highlight a descriptive difference between patient acceptability and clinician confidence in the current model’s clinical utility. This gap is also reflected in the SUS scores: patients reported a higher mean SUS score (M = 70.4, SD = 19.7), indicating good usability, while neuropsychologists reported a substantially lower score (M = 51.7, SD = 20.9), suggesting only marginal usability. In contrast, interaction quality was comparable between the two groups (patients: M = 6.33, SD = 1.03; neuropsychologists: M = 6.67, SD = 1.97), indicating that real-time interpersonal engagement remained largely intact across the digital interface. Overall, these descriptive findings indicate a mismatch between patient acceptability and clinician confidence in the current RCA model. The results are presented in Table 2 and Figure 3. The exploratory inferential statistics are provided in Supplementary Material File S6.

### 3.2. Qualitative Results

#### General Practitioner

Semi-structured qualitative interviews (approximately 30 min) were conducted with four GPs from different primary care practices involved in this pilot study. The aim was to gather experiential data on the implementation, acceptance, challenges and prospects of RCA among older adults. The results focusing on (1) patient acceptance, (2) technical feasibility, (3) interdisciplinary collaboration, (4) implementation barriers, and (5) future perspectives were thematically analyzed and coded. Supplementary Material File S4 (Table S1) provides direct GP quotes to illustrate each subtheme and clarify the sentiment. GPs expressed a positive overall impression of the RCA concept, highlighting its potential to overcome geographic and logistical barriers, especially in rural regions. The possibility of providing cognitive screening and support within a local primary care settings was considered a valuable low threshold offer that could reduce patient travel to specialized memory clinics. When introducing the procedure, patients’ acceptance was generally good (GP3: “*When we offered it, the patients generally accepted it.*”; GP2: “*Because of the clinical question that was already present, the acceptance to do the test was actually high.*”). Occasionally, hesitation from the patients’ side was present or they showed the usual reticence toward testing, regardless of the remote format, often linked to anxiety about cognitive evaluations in general rather than the telemedicine aspects specifically (GP1: “*It is simply the same reluctance people always have with testing*.”). However, patients reported relief after completing the assessment, noting that “*it actually wasn’t that bad*” (GP3). Patients with greater familiarity and comfort with technology tended to engage more easily with the digital format, whereas “*for people who never use a computer, it was more difficult*” (GP3).

The software and technical implementation were reported as straightforward and user-friendly (GP1: “*The software was actually quite simple […] Everything worked really well.*”). The study team was available by telephone for technical support; this was used once for a minor speaker setup issue (GP3: “*Once we had a problem with the speaker*”). No further major technical difficulties were reported. A critical facilitator was the presence of trained personnel to support patients during the remote assessment. This support was essential for patients with advanced cognitive or neuropsychiatric impairments and helped overcome barriers related to unfamiliarity with technology. They emphasized that having staff physically present during testing was important to assist with procedural and technical issues and to provide reassurance, particularly for older patients with limited digital literacy. The communication between GPs and the research and clinical team from the IDMC was described as efficient and “*[…] just as good and just as positive […] just like with other specialists*” (GP2). Written reports were provided promptly (one exception was noted where the report took longer than expected) and comprehensively. Nonetheless, practices noted the potential value of more direct, perhaps telephonic or video-based, follow-up discussions regarding complex cases to better align interpretations and care planning (GP1: “*For more complex cases, an additional discussion would be useful.*”).

A recurrent theme was infrastructural constraints within the practices, notably limited physical space to dedicate to the remote testing setup (GP3: “*The main issue for us is the available rooms.*”), which restricted the number of patients that could be evaluated. Future expansion of the service into routine clinical care would require addressing these spatial limitations. Additionally, clarity in workflow and responsibility, especially in cases where testing might be conducted outside the GP’s office, for example, in collaboration with community pharmacies, was identified as an area for refinement. Ensuring smooth referral pathways, clear indication criteria, and seamless information flow between all parties would be critical. GP practices expressed willingness to continue referring patients for RCA (GP4: “*We could imagine using the platform in the future*”), recognizing its usefulness in their clinical workflow and patient population. The pilot phase was viewed as a promising foundation (GP4: “*Overall it is a good concept*”), with potential for integration into routine primary care given adequate resources and structural support.

## 4. Discussion

This pilot mixed-method study explored the feasibility, usability, and clinical integration of a digitally supported RCA model in general practice, combining structured quantitative surveys from patients and neuropsychologists with qualitative interviews from GPs. The central finding was a clear discrepancy between high acceptability among patients and GPs and more critical neuropsychologist evaluations of usability, diagnostic effectiveness, and clinical confidence. Thus, while the RCA model was technically feasible and well received by patients, the findings also indicate that patient satisfaction alone is insufficient to support routine clinical implementation unless diagnostic confidence and clinician workflow usability are improved. Patient feedback indicates that the RCA model was generally well received. High satisfaction scores for audio and video quality and a strong overall satisfaction rating suggest that the technical aspects of remote assessment were successfully implemented in this older adult cohort. Importantly, patients reported a strong willingness to recommend the procedure, underscoring its acceptability as a complementary pathway for cognitive testing, consistent with prior research suggesting high construct validity and acceptance of digital memory tests [37].

These findings are particularly notable given the demographic challenges of engaging older populations with digital tools. The moderate scores for simplicity and perceived effectiveness suggest variability in user engagement, likely reflective of differing digital literacy levels and cognitive abilities. This reinforces prior findings that older adults’ digital adoption is highly contingent on perceived ease of use and contextual support [38,39]. The low incidence of technical issues further highlights the role of well-supported on-site facilitation, a key factor emphasized by GPs in qualitative interviews. In contrast to the generally positive patient evaluations, neuropsychologists provided a more critical appraisal. Although technical elements, such as audio and video quality, were rated positively, the user interface and simplicity were scored lower, indicating friction in the clinical workflow. Notably, communication and interaction quality were variable, and the perceived effectiveness of the RCA as a clinical tool was rated particularly low, raising concerns about diagnostic depth and clinical confidence.

This divergence represents the major implementation-relevant finding of this study. It shows that successful technical delivery and patient acceptance do not necessarily translate into clinician confidence or perceived clinical utility. For neuropsychologists, the value of RCA depends not only on whether the assessment can be completed, but also on whether the format supports reliable observation, nuanced interaction, diagnostic interpretation, and efficient workflow. Therefore, the lower ratings of effectiveness and usability should not be interpreted as secondary concerns, but as core barriers of routine implementation. While the platform functioned well in facilitating remote interaction, the neuropsychologists’ hesitations underscore that effective cognitive assessment involves nuanced, relational dynamics and real-time clinical judgment, which may be constrained in remote formats [40]. While some studies have validated remote tools for cognitive and Alzheimer’s risk stratification [41,42], the skepticism points to a broader challenge in digital health: ensuring that technological solutions do not compromise clinical richness and diagnostic integrity [43,44].

The value of in-person support during the RCA emerged across all stakeholder perspectives. GPs emphasized that the presence of trained medical personnel was instrumental in supporting older patients, particularly those with advanced cognitive or psychiatric conditions. This human support layer helped overcome barriers associated with low digital literacy and maintained procedural fluency during testing. These insights echo the “human-technology partnership” emphasized in hybrid models of care, where digital tools are enhanced by interpersonal scaffolding [45]. From an implementation perspective, this highlights the importance of integrated care pathways where digital assessments are embedded within well-structured, locally supported workflows. Practices with engaged staff and clearly defined responsibilities reported smoother adoption, consistent with the Normalization Process Theory, which stresses the role of internal coherence and relational work in sustaining new practices [46].

GPs provided a valuable system-level view, confirming the model’s potential to reduce barriers to specialist access, especially in rural or underserved settings. They appreciated being able to initiate early cognitive assessment within a local primary care context, aligning with international policy goals for low-threshold dementia screening [14]. Patient acceptance, according to GPs, was generally positive and mirrored what is often observed in standard assessments, suggesting that digital modality per se was not a major source of resistance. However, operational and infrastructural challenges were evident. Limited physical space and lack of dedicated rooms restricted the number of patients that could be assessed, underscoring the need for logistical planning in any future rollout. GPs also highlighted a need for more dynamic communication with neuropsychologists (e.g., debrief calls for complex cases) to bridge the gap between assessment output and care planning. This signals that while asynchronous reporting (e.g., written reports) is efficient, it may not suffice for nuanced decision-making and continuity of care. Finally, the GPs’ interest in continuing the model speaks to its perceived clinical relevance. However, they stressed that sustainability would depend on workflow clarity, dedicated resources, and cross-sector coordination, especially if the model expands beyond GP practices (e.g., into pharmacies or community centers).

Importantly, the present model should not be interpreted as replacing in-person memory clinic assessment. Face-to-face assessment remains essential for many patients and offers advantages, including a direct rapport and behavioral observation. Rather, GP practice-based RCA should be understood as a complementary access pathway that may support initial evaluation and triage within stepped cognitive care. The primary advantage of this model in the present pilot is reduced travel burden, local accessibility, and the provision of technical support for patients who may not be able to complete home-based digital assessments independently.

Together, this pilot mixed-method study suggests that digitally supported RCA models may be acceptable if clinician-facing barriers are addressed. Their ability to extend specialist input into primary care aligns with stepped care frameworks for dementia and other neurocognitive disorders [10]. However, the findings do not support routine implementation based on patient acceptability alone. The key implementation challenge is the mismatch between high patient and GP acceptance and lower neuropsychologist confidence in diagnostic effectiveness and usability. Future development should therefore prioritize clinician-facing usability, diagnostic confidence and workflow integration before RCA can be considered a scalable component of routine cognitive care pathway.

### Limitations and Future Research

Several limitations should be considered when interpreting the findings of this pilot study. Firstly, the small sample size, in terms of the patient cohort (*n* = 10), neuropsychologists assessment ratings (10 RCAs completed by three neuropsychologists) and the number of general practitioners involved (*n* = 4), limits the generalizability and representativeness of the results. In addition, patients were not recruited consecutively from all GP practices but were referred pragmatically when cognitive concerns were identified by the GP or raised by the patient. This may have introduced selection bias, as included patients may not be representative of all older adults with cognitive concerns in primary care. Similarly, participating GP practices may have been more motivated, better resourced, or more interested in digital innovation than average primary care practices. Therefore, the findings should be interpreted as exploratory pilot data from a specific rural primary care setting rather than as representative of the broader population of older adults, GP practices, or neuropsychologists. A larger and more diverse sample would be needed to validate these findings across different populations and healthcare settings. Secondly, the pilot nature of this study and the limited duration may have influenced the depth of user experience and the ability to fully assess long-term feasibility and clinical outcomes. The novelty of technology and intervention could have introduced a learning curve effect, particularly among patients and staff less familiar with digital tools. Thirdly, the qualitative data were based on interviews with a limited number of GPs, which might not capture the full range of perspectives or challenges encountered in broader clinical practice. Further, the interviews were conducted by the first author, who was involved in this study, introducing the possibility of interviewer and confirmation bias. GPs may have been influenced by the interviewer’s role in the project or may have felt inclined to provide favorable feedback. Although a structured interview guide was used and GPs were asked about both benefits and barriers, the possibility that responses or interpretations were shaped by expectations regarding the RCA model cannot be excluded. Additionally, technical aspects, while generally stable, were evaluated in a controlled pilot context, and scalability issues such as infrastructure constraints and workflow integration remain to be thoroughly examined in routine clinical environments. Lastly, this study did not include a direct comparison group (e.g., traditional in-person cognitive assessments). Therefore, this study does not assess diagnostic validity or diagnostic accuracy. The findings regarding diagnostic usefulness are limited to stakeholder perceptions and should not be interpreted as evidence of diagnostic validity or reliability. Additionally, RCA was not offered in the patients’ homes; therefore, the findings cannot determine whether assessment in a GP practice is preferable to, equivalent to, or less acceptable than home-based RCA or in-person memory clinic assessment. Future studies with larger samples, longer follow-up, and comparative designs are warranted to comprehensively evaluate efficacy, cost effectiveness, and implementation strategies for remote cognitive assessment in primary care.

## 5. Conclusions

This pilot mixed-method study demonstrates that a digitally supported Remote Cognitive Assessment (RCA) model is technically feasible and generally well accepted by older patients and GPs in the primary care setting. High patient satisfaction and the successful delivery of assessments with minimal technical issues highlight the potential of RCA to increase accessibility, particularly in underserved or rural areas. However, the low ratings from neuropsychologists, especially regarding diagnostic effectiveness and usability, underline important limitations in clinical utility and show a major mismatch between high patient satisfaction and neuropsychologists’ critical clinical point of view, which must be addressed. Patient satisfaction is therefore insufficient to justify routine clinical implementation. Determining whether such models can be successful in routine care will require evidence beyond feasibility and acceptability, including diagnostic accuracy, workflow burden, staffing, infrastructure, reimbursement, and cost effectiveness. RCA may help expand access to cognitive diagnostics in underserved or rural areas, but only if it can be integrated in a way that meets both patient access needs and clinical standards. Therefore, these findings should be interpreted as preliminary feasibility evidence rather than evidence for scalable implementation. Future development must therefore focus on improving diagnostic confidence, clinician workflow usability and the appropriate infrastructure needed to support reliable assessment.

## Figures and Tables

**Table 1:** Demographic characteristics.

Characteristic (*n* = 10)	M		SD	
Age	77.03		14.1	
MoCA ^1^	19.25		2.67	
GDS ^2^	2.67		1.63	
Education, in years	10.5		1.77	
Distance to GP ^3^, in km	3.15		2.87	
Travel time to GP by public transport *, in hh:mm	00:08		0.003	
Distance to IDMC Bern ^4^, in km	54.32		33.75	
Travel time to IDMC Bern by public transport *, in hh:mm	01:41		0.03	
Distance GPs to IDMC Bern, in km	51.25		31.46	
Age range	Min.		Max.	
	55		89	
Gender	Female		Male	
	4 (40%)		6 (60%)	
Prior technology use	Yes		No	
	9 (90%)		1 (10%)	
Living situation **	Rural		Urban	
	10 (100%)		0 (0%)	
Referral reason	Self-concerns		GP-concerns	
	2 (20%)		8 (80%)	
Suspected cognitive status after RCA	Healthy	MCI ^5^		AD ^6^
	5 (50%)	3 (30%)		2 (20%)
Referral for in-person memory clinic appointment based on RCA results	Yes		No	
	5 (50%)		5 (50%)	

^1^ Montreal Cognitive Assessment. ^2^ Geriatric Depression Scale. ^3^ General practitioner. ^4^ Interdisciplinary Memory Clinic Bern. ^5^ Mild Cognitive Impairments. ^6^ Alzheimer’s disease. * Travel time was calculated in hours using the best available public transport option during normal working hours. ** Living situation is based on the Statistical Cities 2020 dataset from the Federal Statistical Office Switzerland [36].

**Table 2:** Side-by-side comparison of user experience from patients and neuropsychologists during remote cognitive assessments.

Evaluation Criteria	Patients (*n* = 10)			Neuropsychologists (10 RCAs ^1^ Completed by Three Neuropsychologists)		
	M	SD	Med	M	SD	Med
Audio Quality	8	2.28	8.5	8.17	1.17	8.0
Communication	7.33	0.82	7.5	3.83	3.06	2.5
Effectiveness	5.83	2.14	6.5	2.83	2.71	2.0
Interaction	6.33	1.03	6.0	6.67	1.97	6.0
Overall Satisfaction	8.33	0.82	8.5	4.33	2.5	3.5
Recommending to Others	8.83	1.17	9.0	4.67	1.63	5.0
Simplicity	5	3.41	4.5	3.33	1.86	3.0
User Interface	7.67	1.51	8.0	5.67	2.16	6.0
Video Quality	9.17	1.17	9.5	8	1.67	8.0
SUS-Score ^2^	70.4	19.7	68.8	51.7	20.9	53.8
Technical Problems (reverse coded)	1.67	0.82	1.5	3.83	2.56	4.0

Values represent mean ratings (M), standard deviation (SD) and median (Med) for each evaluation criterion and SUS scores. Higher scores indicate more positive evaluations, except for technical problems, where higher values reflect more reported issues. ^1^ Remote Cognitive Assessment. ^2^ System Usability Scale Score.

## Data Availability

The data and analytical code supporting the findings of this study are available from the corresponding author (A.H.) on reasonable request due to privacy reasons.

## Supplementary Materials

The following supporting information can be downloaded at: https://www.mdpi.com/article/10.3390/healthcare14111452/s1, Supplementary Material File S1—Questionnaire S1: User Experience Patients; Supplementary Material File S2—Questionnaire S2: User Experience Neuropsychologists; Supplementary Material File S3—Questionnaire S3: System Usability Scale; Supplementary Material File S4—Table S1: Thematic coding framework with illustrative general practitioner (GP) quotes; Supplementary Material File S5—Figure S1: Participant and Data Flow Diagram; Supplementary Material File S6—Table S2: Side-by-side comparison of user experience from patients and neuropsychologists during remote cognitive assessments.

## Abbreviations

The following abbreviations are used in this manuscript:

AD	Alzheimer’s Disease
BNT-15	Boston Naming Task
CERAD	Consortium to Establish a Register for Alzheimer’s Disease
GDS-15	Geriatric Depression Scale
GP	General practitioner
IDMC	Interdisciplinary Memory Clinic
M	Mean
Med	Median
MoCA	Montreal Cognitive Assessment
OECD	Organisation for Economic Co-operation and Development
RCA	Remote Cognitive Assessment
SD	Standard Deviation
SUS	System Usability Scale
USE	User Satisfaction and Ease of use
VC	Videoconference
