# Supplementary material for: Video-Supported Remote Cognitive Assessment in General Practice—A Pilot Mixed-Method Study on Usability, Acceptability and Feasibility
Source: Healthcare (Basel). 2026 May 25;14(11):1452. doi: 10.3390/healthcare14111452 (PMC13257314; doi:10.3390/healthcare14111452)
Supplement: Supplementary file 1 [file healthcare-14-01452-s001.zip › S1_UserExperience_Patients.pdf]

UNIVERSITÄT BERN

UNIVERSITÄRE PSYCHIATRISCHE DIENSTE BERN

VP-Nr.___________________________

Datum _____/_______/_______ (TT/MM/JJJJ) Uhrzeit: _________:___________Uhr

Nutzererfahrung Patient/innen

Vielen Dank für Ihre Teilnahme an unserem Pilotprojekt zur «Remote Memory Clinic». Ihr wertvolles Feedback zu technischen Aspekten, Nutzererfahrung und Effektivität der Fernuntersuchung ist von grosser Bedeutung, um die Qualität zu verbessern.

Wie einfach war es für Sie, die Videotelefonie-Software zu verwenden?

1 2 3 4 5 6 7 8 9 10

Sehr schwer — Mittelmässig — Sehr einfach

Gab es während der Nutzung technische Probleme oder Schwierigkeiten?

1 2 3 4 5 6 7 8 9 10

Überhaupt nicht — Sehr viele

Wie bewerten Sie die Qualität der Videoverbindung während der Testung?

1 2 3 4 5 6 7 8 9 10

Sehr schlecht — Hervorragend

Wie bewerten Sie die Qualität der Audioverbindung während der Testung?

1 2 3 4 5 6 7 8 9 10

Sehr schlecht — Hervorragend

Hatten Sie das Gefühl, dass die Untersuchung aus der Ferne genauso effective war wie eine persönliche Untersuchung?

1 2 3 4 5 6 7 8 9 10

Schlechter — Gleich — Besser

Wie würden Sie die Nutzeroberfläche der Videotelefonie-Software bewerten?

1	2	3	4	5	6	7	8	9	10

Sehr nutzerunfreundlich Sehr nutzerfreundlich

Welche Funktion der Videotelefonie-Software haben Sie besonders geschätzt?

Gibt es Verbessungsvorschläge bezüglich der Nutzerfreundlichkeit?

Wie würden Sie die Kommunikation und Verständnis während der Testungen beurteilen?

1 2 3 4 5 6 7 8 9 10

Sehr schlecht Hervorragend

Wie gut konnten Sie mit dem/der Neuropsycholog/in während der Testung interargieren?

1 2 3 4 5 6 7 8 9 10

Sehr schlecht Sehr gut

Wie zufrieden sind Sie insgesamt mit der «Remote Memory Clinic»?

1 2 3 4 5 6 7 8 9 10

Überhaupt nicht Sehr zufrieden

Würden Sie die «Remote Memory Clinic» Freund/innen und Familienmitgliedern weiterempfehlen?

1 2 3 4 5 6 7 8 9 10

Nein Vielleicht Ja

Haben Sie zusätzliche Kommentare oder Anmerkungen, die Sie gerne teilen möchten?

____

____

____

____

____

____

____

UNIVERSITÄT BERN
UNIVERSITÄRE PSYCHIATRISCHE DIENSTE BERN

VP-Nr.___________________________

Datum _____/_______/_______ (TT/MM/JJJJ) Uhrzeit: __________:____________Uhr

User Experience Patients (English translation)

Thank you very much for your participation in our pilot project on the “Remote Memory Clinic.” Your valuable feedback on technical aspects, user experience, and the effectiveness of the remote assessment is of great importance in helping us improve quality.

How easy was it for you to use the videoconference system?

1 2 3 4 5 6 7 8 9 10

Very difficult | Moderately | Very easy

Did you experience any technical problems or difficulties during use?

1 2 3 4 5 6 7 8 9 10

Not at all | A lot

How would you rate the quality of the video connection during the assessment?

1 2 3 4 5 6 7 8 9 10

Very poor | Excellent

How would you rate the quality of the audio connection during the assessment?

1 2 3 4 5 6 7 8 9 10

Very poor | Excellent

Did you feel that the remote assessment was as effective as an in-person assessment?

1 2 3 4 5 6 7 8 9 10

Worse | Same | Better

How would you rate the user interface of the videoconference software?

1 2 3 4 5 6 7 8 9 10

Very userunfriendly Very userfriendly

Which function of the videoconference system did you particularly appreciate?

Do you have any suggestions for improving user-friendliness?

How would you rate the communication and understanding during the assessment?

1	2	3	4	5	6	7	8	9	10

Very poor Excellent

How well were you able to interact with the neuropsychologist during the assessment?

1	2	3	4	5	6	7	8	9	10

Very poorly Very well

How satisfied are you overall with the «Remote Memory Clinic»?

1	2	3	4	5	6	7	8	9	10

Not at all Very satisfied

Would you recommend the «Remote Memory Clinic» to friends or family members?

1	2	3	4	5	6	7	8	9	10

No Maybe Yes

Do you have any additional comments or remarks you would like to share?
