# Supplementary material for: Video-Supported Remote Cognitive Assessment in General Practice—A Pilot Mixed-Method Study on Usability, Acceptability and Feasibility
Source: Healthcare (Basel). 2026 May 25;14(11):1452. doi: 10.3390/healthcare14111452 (PMC13257314; doi:10.3390/healthcare14111452)
Supplement: Supplementary file 1 [file healthcare-14-01452-s001.zip › S2_UserExperience_Neuropsychologists.pdf]

b UNIVERSITÄT BERN

UNIVERSITÄRE PSYCHIATRISCHE DIENSTE BERN

VP-Nr.___________________________

Datum ____/______/______ (TT/MM/JJJJ) Uhrzeit: _________:___________Uhr

Nutzererfahrung Memory Clinic

Vielen Dank für Ihre Teilnahme an unserem Pilotprojekt zur «Remote Memory Clinic». Ihr wertvolles Feedback zu technischen Aspekten, Nutzererfahrung und Effektivität der Fernuntersuchung ist von grosser Bedeutung, um die Qualität zu verbessern.

Wie einfach war es für Sie, die Videotelefonie-Software zu verwenden?

1 2 3 4 5 6 7 8 9 10

Sehr schwer | Mittelmässig | Sehr einfach

Gab es während der Nutzung technische Probleme oder Schwierigkeiten?

1 2 3 4 5 6 7 8 9 10

Überhaupt nicht | Sehr viele

Wie bewerten Sie die Qualität der Videoverbindung während der Testung?

1 2 3 4 5 6 7 8 9 10

Sehr schlecht | Hervorragend

Wie bewerten Sie die Qualität der Audioverbindung während der Testung?

1 2 3 4 5 6 7 8 9 10

Sehr schlecht | Hervorragend

Hatten Sie das Gefühl, dass die Untersuchung aus der Ferne genauso effective war wie eine persönliche Untersuchung?

1	2	3	4	5	6	7	8	9	10

Schlechter Gleich Besser

Wie würden Sie die Nutzeroberfläche der Videotelefonie-Software bewerten?

1	2	3	4	5	6	7	8	9	10

Sehr nutzerunfreundlich Sehr nutzerfreundlich

Welche Funktion der Videotelefonie-Software haben Sie besonders geschätzt?

Gibt es Verbessungsvorschläge bezüglich der Nutzerfreundlichkeit?

Wie würden Sie die Kommunikation oder Verständnis während der Testungen bewerten?

1 2 3 4 5 6 7 8 9 10

Sher schlecht Sehr gut

Wie gut konnten Sie mit dem/der Patient/in während der Testung interargieren?

1 2 3 4 5 6 7 8 9 10

Sehr schlecht Sehr gut

Wie zufrieden sind Sie insgesamt mit der «Remote Memory Clinic»?

1 2 3 4 5 6 7 8 9 10

Überhaupt nicht Sehr zufrieden

Würden Sie die «Remote Memory Clinic» anderen Neuropsycholog/innen weiterempfehlen?

1 2 3 4 5 6 7 8 9 10

Nein Vielleicht Ja

Haben Sie zusätzliche Kommentare oder Anmerkungen, die Sie gerne teilen möchten?

____

____

____

____

____

____

____

VP-Nr.___________________________

Datum _____/_______/_______ (TT/MM/JJJJ)   Uhrzeit: __________:____________Uhr

User Experience Memory Clinic (English translation)

Thank you very much for your participation in our pilot project on the "Remote Memory Clinic." Your valuable feedback on technical aspects, user experience, and the effectiveness of the remote assessment is of great importance in helping us improve quality.

How easy was it for you to use the videoconference system?

1 2 3 4 5 6 7 8 9 10

Very difficult   Moderately   Very easy

Did you experience any technical problems or difficulties during use?

1 2 3 4 5 6 7 8 9 10

Not at all   A lot

How would you rate the quality of the video connection during the assessment?

1 2 3 4 5 6 7 8 9 10

Very poor   Excellent

How would you rate the quality of the audio connection during the assessment?

1 2 3 4 5 6 7 8 9 10

Very poor   Excellent

Did you feel that the remote assessment was as effective as an in-person assessment?

1	2	3	4	5	6	7	8	9	10

Worse Same Better

How would you rate the user interface of the videoconference software?

1	2	3	4	5	6	7	8	9	10

Very userunfriendly Very userfriendly

Which function of the videoconference system did you particularly appreciate?

Do you have any suggestions for improving user-friendliness?

How would you rate the communication and understanding during the assessment?

1	2	3	4	5	6	7	8	9	10

Very poor Excellent

How well were you able to interact with the patient during the assessment?

1	2	3	4	5	6	7	8	9	10

Very poorly Very well

How satisfied are you overall with the «Remote Memory Clinic»?

1	2	3	4	5	6	7	8	9	10

Not at all Very satisfied

Would you recommend the «Remote Memory Clinic» to other neuropsychologists?

1	2	3	4	5	6	7	8	9	10

No Maybe Yes

Do you have any additional comments or remakrs you would like to share?
