# Supplementary material for: Video-Supported Remote Cognitive Assessment in General Practice—A Pilot Mixed-Method Study on Usability, Acceptability and Feasibility
Source: Healthcare (Basel). 2026 May 25;14(11):1452. doi: 10.3390/healthcare14111452 (PMC13257314; doi:10.3390/healthcare14111452)
Supplement: Supplementary file 1 [file healthcare-14-01452-s001.zip › S3_SUS.pdf]

UNIVERSITÄT BERN

UNIVERSITÄRE PSYCHIATRISCHE DIENSTE BERN

VP-Nr.___________________________

Datum _____/_______/_______ (TT/MM/JJJJ) Uhrzeit: _________:___________Uhr

Fragebogen zur System Gebräuchlichkeit

Vielen Dank für Ihre Teilnahme an unserem Pilotprojekt zur «Remote Memory Clinic». Ihr wertvolles Feedback zur System Gebräuchlichkeit ist von grosser Bedeutung, um die Qualität zu verbessern.

Ich denke, dass ich das System gerne häufig nutzen würde.

1 2 3 4 5

Stimme überhaupt nicht zu — Stimme voll zu

Ich fand das System unnötig komplex.

1 2 3 4 5

Stimme überhaupt nicht zu — Stimme voll zu

Ich fand das System einfach zu bedienen.

1 2 3 4 5

Stimme überhaupt nicht zu — Stimme voll zu

Ich glaube, ich würde Unterstützung von einer technisch versierten Person benötigen, um das System nutzen zu können.

1 2 3 4 5

Stimme überhaupt nicht zu — Stimme voll zu

Ich fand, dass die Funktionen in diesem System gut integriert waren.

1 2 3 4 5

Stimme überhaupt nicht zu — Stimme voll zu

Ich denke, dass das System zu viel Inkonsistenz aufwies.

1 2 3 4 5

Stimme überhaupt nicht zu — Stimme voll zu

Ich kann mir vorstellen, dass die meisten Menschen den Umgang mit diesem System sehr schnell lernen.

1 2 3 4 5

Stimme überhaupt nicht zu — Stimme voll zu

Ich fand das System sehr umständlich zu bedienen.

1 2 3 4 5

Stimme überhaupt nicht zu — Stimme voll zu

Ich fühlte mich bei der Benutzung des Systems sehr sicher.

1 2 3 4 5

Stimme überhaupt nicht zu — Stimme voll zu

Ich musste erst eine Reihe von Dingen lernen, bevor ich mit dem System umgehen konnte.

1 2 3 4 5

Stimme überhaupt nicht zu — Stimme voll zu

VP-Nr.____________________________

Datum _____/_______/_______ (TT/MM/JJJJ) Uhrzeit: _________:___________Uhr

System Usability Scale (English translation)

Thank you very much for your participation in our pilot project on the “Remote Memory Clinic.” Your valuable feedback on system usability of the remote assessment is of great importance in helping us improve quality.

I think that I would like to use this system frequently.

1 2 3 4 5

Strongly disagree Strongly agree

I found the system unnecessarily complex.

1 2 3 4 5

Strongly disagree Strongly agree

I thought the system was easy to use.

1 2 3 4 5

Strongly disagree Strongly agree

I think that I would need the support of a technical person to be able to use this system.

1 2 3 4 5

Strongly disagree Strongly agree

I found the various functions in this system were well integrated.

1 2 3 4 5

Strongly disagree Strongly agree

I thought there was too much inconsistency in this system.

1 2 3 4 5

Strongly disagree Strongly agree

I would imagine that most people would learn to use this system very quickly.

1 2 3 4 5

Strongly disagree Strongly agree

I found the system very cumbersome to use.

1 2 3 4 5

Strongly disagree Strongly agree

I felt very confident using the system.

1 2 3 4 5

Strongly disagree Strongly agree

I needed to learn a lot of things before I could get going with this system.

1 2 3 4 5

Strongly disagree Strongly agree
