# Supplementary material for: Video-Supported Remote Cognitive Assessment in General Practice—A Pilot Mixed-Method Study on Usability, Acceptability and Feasibility
Source: Healthcare (Basel). 2026 May 25;14(11):1452. doi: 10.3390/healthcare14111452 (PMC13257314; doi:10.3390/healthcare14111452)
Supplement: Supplementary file 1 [file healthcare-14-01452-s001.zip › S4_GP quotes.pdf]

Table S1. Thematic coding framework with illustrative general practitioner (GP) quotes

Theme	Code	Selected GPs quotes
Patient Acceptance	Acceptance	GP2: "Because of the clinical question that was already present, the acceptance to do the test was actually high."<br>GP2: "The patients already had a prior history of memory problems, so they wanted to see what the result would be."<br>GP3: "When we offered it, the patients generally accepted it."<br>GP4: "There were actually no particular concerns about it being online."
	General hesitation	GP1: "The same hesitation as always with these kinds of tests."<br>GP1: "It is no different than when we offer a MoCA or MMSE here in the practice."<br>GP1: "It is simply the same reluctance people always having with testing."<br>GP2: "I have the feeling they (patients) come as it is were an exam."<br>GP3: "They (patients) are simply very tense"<br>GP4: "Not more or not less than with other memory tests."
	Familiarity with technology	GP1: "Patients with a certain affinity for technology had fewer difficulties."<br>GP1: "It depends on whether they use computers or smartphones in everyday life."<br>GP3: "For people who never use a computer, it was more difficult."<br>GP2: "With more advanced condition it was more demanding."
	Relief	GP3: "At the end they said it actually wasn't that bad."<br>GP3: "Afterwards some said they had imagined it to be worse."<br>GP4: "It was a relief once they were finished"
Technical Feasibility	Software usability	GP1: "The software was actually quite simple."<br>GP1: "Everything worked really well."<br>GP1: "The procedure was basically self-explanatory"<br>GP2: "The operation was uncomplicated."<br>GP3: "Overall the platform worked well."<br>GP4: "We didn't have any issues with the platform. Everything worked well."
	Instruction manuals	GP1: "The manual was very well described, step by step."<br>GP2: "The instructions were clearly described."<br>GP2: "The instructions were very clearly structured."

		GP3: "The folder was really excellent." GP4: "The manual was really helpful, you didn't have to invest much time in preparation"
	On-site assistance	GP2: "It was good that someone was in the room who could help." GP3: "Without someone there, it would have been more difficult." GP4: "If someone was present, it worked well."
	Technical issues	GP3: "Once we had a problem with the speakers" GP3: "At the beginning we had to adjust the microphone ones." GP3: "Occasionally there were small technical adjustments needed."
Interdisciplinary Collaboration	Communication	GP2: "The collaboration was just like with other specialists." GP2: "It (communication) was just as good and just as positive." GP3: "If we had a problem, we could call and reach someone." GP3: "Communication worked really well." GP3: "It was helpful that we could contact someone directly." GP4: "We could always ask questions."
	Potential for deeper discussion	GP1: "For more complex cases, an additional discussion would be useful." GP1: "In difficult cases it would be helpful to exchange impressions." GP2: "Maybe it would be helpful to briefly discuss the findings by phone."
Implementation Barriers	Infrastructure limitations	GP1: "At the moment we have a space problem." GP1: "We don't have many rooms, so it is difficult to let someone sit there for that long." GP2: "If we had more space, we would definitely use the service." GP3: "The main issue for us is the available rooms."
	Written reports	GP3: "Once the report arrived quite late." GP4: "Written reports were easy to read and helped in communicating further procedure with patients."
Future Perspectives	Need for clear workflows	GP1: "The process would need to be clearly defined." GP1: "The information flow would need to be well organized." GP2: "You would need clear referral pathways."

	Willingness	GP1: "I would definitely use the service."<br>GP1: "The low-threshold opportunity to have a cognitive test is very valuable. Not having to come to you, but being able to do it here in the practice"<br>GP2: "I would continue referring patients."<br>GP3: "For screenings memory problems, this makes sense and would use it in the future."<br>GP4: "We could imagine using the platform in the future."<br>GP4: "Overall it is a good concept."
