# Supplementary material for: Video-Supported Remote Cognitive Assessment in General Practice—A Pilot Mixed-Method Study on Usability, Acceptability and Feasibility
Source: Healthcare (Basel). 2026 May 25;14(11):1452. doi: 10.3390/healthcare14111452 (PMC13257314; doi:10.3390/healthcare14111452)
Supplement: Supplementary file 1 [file healthcare-14-01452-s001.zip › S5_FlowDiagram.pdf]

Supplementary Material S5

Figure S1. Participant and Data Flow Diagram.

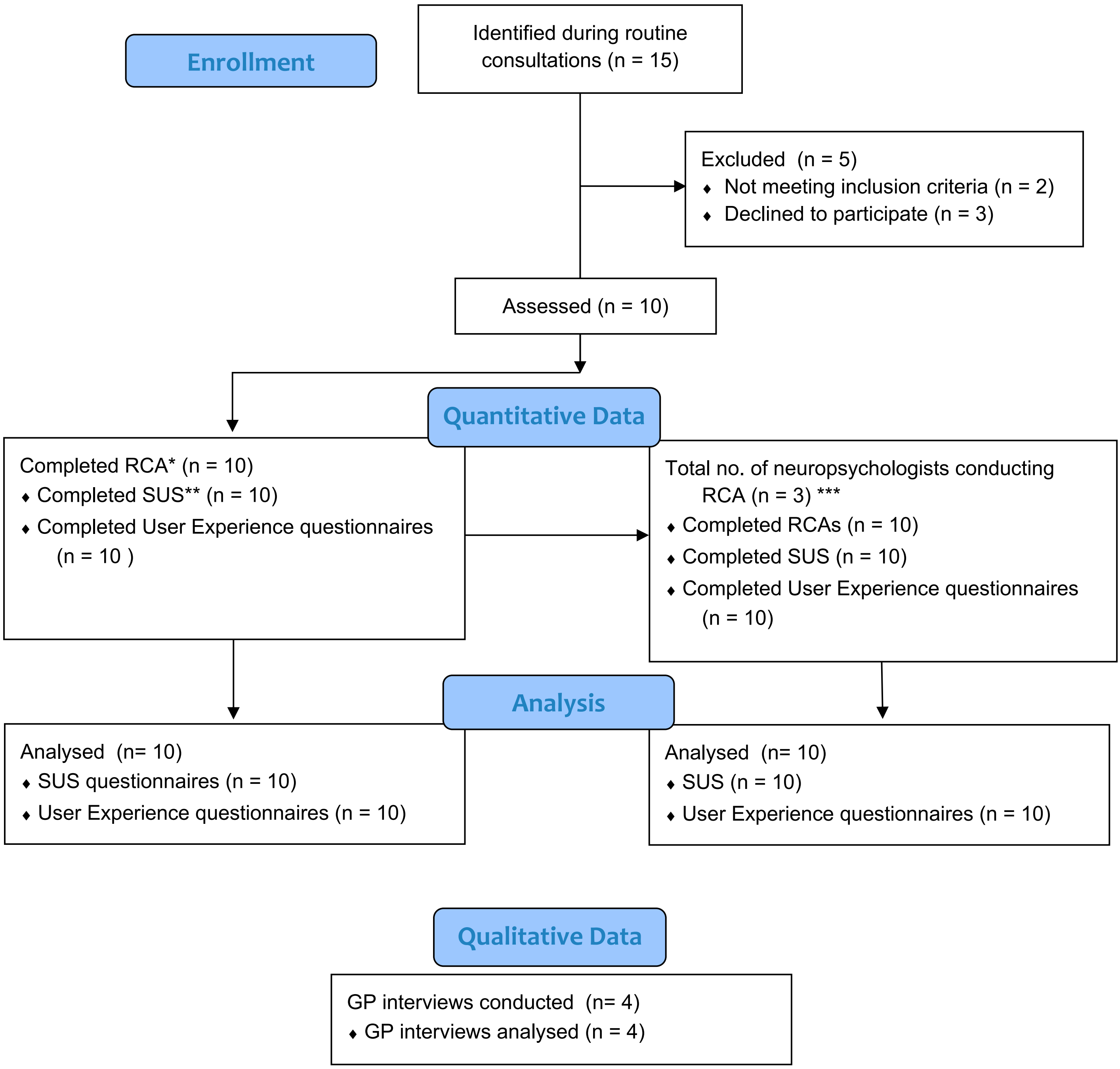

*Remote Cognitive Assessment

**System Usability Scale

***In total, 10 RCAs were conducted by three neuropsychologists. Following each RCA, the questionnaires were completed by the neuropsychologist who had conducted the RCA.
