# Supplementary material for: Video-Supported Remote Cognitive Assessment in General Practice—A Pilot Mixed-Method Study on Usability, Acceptability and Feasibility
Source: Healthcare (Basel). 2026 May 25;14(11):1452. doi: 10.3390/healthcare14111452 (PMC13257314; doi:10.3390/healthcare14111452)
Supplement: Supplementary file 1 [file healthcare-14-01452-s001.zip › S6_ExploratoryInferantialStatistics.pdf]

Supplementary Material File S6

Table S2. Side-by-side comparison of user experience from patients and neuropsychologists during remote cognitive assessments.

**Evaluation Criteria**	**Patients (*n* = 10)**			**Neuropsychologists (*n* = 10;** completed by three neuropsychologists**)**					
	**M**	**SD**	**Med**	**M**	**SD**	**Med**	***W*** [2]	***p*-value**	***r*** [3]
Audio Quality	8	2.28	8.5	8.17	1.17	8.0	16.5	0.87	0.07
Communication	7.33	0.82	7.5	3.83	3.06	2.5	6.5	0.07	0.54
Effectiveness	5.83	2.14	6.5	2.83	2.71	2.0	7.5	0.1	0.49
Interaction	6.33	1.03	6.0	6.67	1.97	6.0	17.5	1	0.02
Overall Satisfaction	8.33	0.82	8.5	4.33	2.5	3.5	4.5	**0.03**	0.64
Recommending to Others	8.83	1.17	9.0	4.67	1.63	5.0	0.5	**0.01**	0.82
Simplicity	5	3.41	4.5	3.33	1.86	3.0	13.5	0.52	0.21
User Interface	7.67	1.51	8.0	5.67	2.16	6.0	7	0.09	0.52
Video Quality	9.17	1.17	9.5	8	1.67	8.0	9.5	0.19	0.41
SUS-Score [1]	70.4	19.7	68.8	51.7	20.9	53.8	11.5	0.34	0.3
Technical Problems **(reverse coded)**	1.67	0.82	1.5	3.83	2.56	4.0	26.5	0.18	0.41

Note. Values represent mean ratings (M), standard deviations (SD) and median (Med) for each evaluation criterion and SUS scores. Higher scores indicate more positive evaluations, except for Technical Problems, where higher values reflect more reported issues. [1] System Usability Scale Score [2] Wilcoxon rank-sum test statistic [3] Rank-biserial correlation.
